# Towards Accurate Diagnosis of Skin Lesions Using Feedforward Back Propagation Neural Networks

**DOI:** 10.3390/diagnostics11060936

**Published:** 2021-05-22

**Authors:** Simona Moldovanu, Cristian-Dragos Obreja, Keka C. Biswas, Luminita Moraru

**Affiliations:** 1 Faculty of Automation, Computers, Electrical Engineering and Electronics, Dunarea de Jos University of Galati, 47 Domneasca Str., 800008 Galati, Romania; simona.moldovanu@ugal.ro; 2 The Modelling & Simulation Laboratory, Dunarea de Jos University of Galati, 47 Domneasca Str., 800008 Galati, Romania; Cristian.obreja@ugal.ro; 3 Faculty of Engineering, Dunarea de Jos University of Galati, 47 Domneasca Str., 800008 Galati, Romania; 4 Department of Biological Sciences, University of Alabama at Huntsville, Huntsville, AL 35899, USA; kcb0015@uah.edu; 5 Faculty of Sciences and Environment, Dunarea de Jos University of Galati, 47 Domneasca Str., 800008 Galati, Romania

**Keywords:** melanoma, non-melanoma, asymmetry, feedforward neural networks, classification, architecture optimization

## Abstract

In the automatic detection framework, there have been many attempts to develop models for real-time melanoma detection. To effectively discriminate benign and malign skin lesions, this work investigates sixty different architectures of the Feedforward Back Propagation Network (FFBPN), based on shape asymmetry for an optimal structural design that includes both the hidden neuron number and the input data selection. The reason for the choice of shape asymmetry was based on the 5–10% disagreement between dermatologists regarding the efficacy of asymmetry in the diagnosis of malignant melanoma. Asymmetry is quantified based on lesion shape (contour), moment of inertia of the lesion shape and histograms. The FFBPN has a high architecture flexibility, which indicates it as a favorable tool to avoid the over-parameterization of the ANN and, equally, to discard those redundant input datasets that usually result in poor test performance. The FFBPN was tested on four public image datasets containing melanoma, dysplastic nevus and nevus images. Experimental results on multiple benchmark data sets demonstrate that asymmetry *A*2 is a meaningful feature for skin lesion classification, and FFBPN with 16 neurons in the hidden layer can model the data without compromising prediction accuracy.

## 1. Introduction

Malignant melanoma is a major public health concern and is one of the deadliest forms of skin cancer. Statistics indicate melanoma as one of the world’s fastest-growing cancers [1,2,3,4]. The cost of medical treatment exceeds $300 million US. Therefore, timely diagnosis of melanoma is critical, and consistent actions to develop models for real-time melanoma detection are needed [5]. An early diagnosis using immunotherapy and targeted therapy was shown to lead to a significant improvement in melanoma treatments [6].

The visual inspection of dermoscopy images, i.e., ABCDE (Asymmetry, Border irregularity, Color variegation, Diameter, Evolution) technique, is a common tool for dermatologists, but it suffers from clinician subjectivity. Often it requires invasive biopsy to confirm the diagnosis, even in a nevus case. The case of dysplastic nevi is more complicated, as this skin lesion shows image features between nevi and melanoma. These are the morphological and biological intermediates between these two entities [7,8]. Dysplastic nevi are larger and irregular in shape compared to an average mole.

The ability to classify the melanoma/skin cancer from other skin diseases belongs to experienced physicians. The Internet of Medical Things (IoMT) and computer-based skin lesion detection could provide recommendations for specialized and non-specialized users, equally. For years, numerous studies have been devoted to the early diagnosis of melanoma using various computational methods. Machine Learning techniques as part of Computer-Aided Diagnosis have been applied successfully for the detection of polyps in colonoscopy [9,10], calcifications in mammography [11], chest imaging [12], automated solutions for melanoma diagnosis using dermoscopic images [13,14,15] and non-invasive methods of recognition of the finger skin [16]. In another approach, the features extracted from the pixels of a lesion were handled by a Stack-Based Auto-Encoder, and various classification methods like Principal Component Analysis, Recurrent Neural Networks and a Softmax Linear Classifier were utilized for automatic diagnosis of pigmented skin lesions [17]. Inevitably, there are limitations, mainly due to the absence of ground truth information to judge the performance of the classifiers concerning the *Sensitivity*, *Specificity*, and *Accuracy* [18,19]. Artificial Neural Networks (ANNs) have progressed significantly in recent years. ANNs have the potential to predict the medical outcome of different kinds of skin lesions. ANNs process data sequentially through a series of layers and aggregate large-scale datasets for training/learning purposes [20,21,22]. A CAD system conceived to discriminate melanoma from nevus based on handcraft ABCDE features using a Mutual Information metric was proposed for a binary classification decision [23].

For these reasons, an extensive analysis was conducted, aiming at assessing the effectiveness of the complex task of automatic diagnosis of melanoma and aiding dermatologists in decision-making. Automatic image analysis is mainly concerned with the identification of dermoscopic features in each image and subsequently using the associations of these relevant features to form a correct diagnosis. This paper also focuses on the fact that the performance of an artificial network highly depends on the stability of the outputs, i.e., that the networks are not over-trained or under-trained.

To achieve these objectives, an exhaustive investigation on efficiency of skin cancer detection with ANNs using shape asymmetry, as a handcrafted feature, was performed. An accurate recognition and discrimination between melanoma and non-melanoma lesions is a demanding task, mainly due to the visual similarity between melanoma and non-melanoma lesions. In addition, the goal of the proposed technique is the simplicity of application by non-experienced physicians. The skin lesion asymmetry is determined by the different methods that were employed, including lesion shape (contour), moment of inertia of the lesion shape and histograms. The reason for the choice of shape asymmetry was based on the 5–10% disagreement between dermatologists regarding the efficacy of the asymmetry in the diagnosis of malignant melanoma. This feature tried to imitate the clinical skin disease diagnosis procedure, which is primarily done by observation. The asymmetry of the lesions has a much greater impact for visual information acquisition. Prior to classification, the GraphCut algorithm for segmentation of skin lesions is utilized. It facilitates the asymmetry computation as a representative feature. Moreover, any other possible features that characterize the surrounding normal tissue are removed from the analysis.

For data classification, the neural network methods are more useful and powerful alternatives to statistical techniques, mainly in the field of regression analysis, classification or probability density estimation [24]. The main advantages of ANNs and, in particular, of the FFBPN, are properties such as self-learning, adaptability, robustness, the accuracy in identifying melanoma in dermoscopic images of lesions, the execution speed and reasonable computation and memory costs. Generally, these advantages are lost when the size of the network increases.

A three-layer Feedforward Back Propagation Network (FFBPN) with one hidden layer is used as a classifier. The main goal of this paper is to optimize this network architecture by working on the selection of the training dataset and the number of hidden neurons. If the selected training dataset does not contain representative samples, then the ANN cannot learn the process properly. The number of hidden neurons determines how well a problem can be learned. The models are trained using one loss function, different combinations of the input modalities (different training datasets) and different numbers of hidden neurons to allow the studied models to be robust to missing data during the testing phase. The number of hidden neurons should be large enough for the correct approach to the problem and low enough for a good generalization capacity. So far, there is no universally accepted method to estimate the optimal size for the hidden layers. The optimality criteria could be determined only for a particular problem under consideration, so the optimal and minimal neural network architectures are strictly related to a given situation. The classification is done using four statistically significant asymmetry features as input data. The network is trained using scaled conjugate gradient backpropagation. A large-scale investigation was conducted, where FFBPN models were investigated on four datasets of dermoscopic and non-dermoscopic images (7-Point, Med-node, PAD-UFES-20 and PH2) for four features. As a result of this work, we provide a guideline for the proper selection of an ANN that can substantially increase the predictive performance in skin lesion detection and classification. In addition, we assess the possible redundancy of the asymmetry feature extracted using four methods.

This paper is organized as follows. Section 2 presents a literature review of the state of the art on skin lesion image segmentation and classification using deep learning approaches; Section 3 presents image datasets for melanoma diagnosis accessible via the internet and describes the FFBPN architectures and the implementation details; Section 4 discusses the experimental results; finally, Section 5 gives some concluding remarks.

## 2. Related Works

ANNs have been used in the past to accurately predict pulmonary diseases such as pneumonia, lung nodules or cardiac arrhythmia [25,26]. Accurate and representative features of equivocal skin lesion images improve diagnosis and are pivotal in the classification of melanoma. These representative features enhance the performance of the ANN in the diagnosis of doubtful skin lesions. Skin lesion asymmetry is a strong and efficient feature in the differentiation between benign and malign skin lesions [22], i.e., the asymmetric degree of a skin lesion is an intuitive mark of its deadly potential. Shape asymmetry mathematically models the human observation of a lesion and correlates it to the ABCD rule for lesion classification. Global and local texture characteristics, i.e., GLCM parameters and SURF features, were used for classification of melanoma by the instrumentality of SVM and KNN algorithms [27]. The authors reported an accuracy of 79.3% and 78.2% using SVM and KNN for GLCM parameters, and of 87.3% and 85.2% using SVM and KNN for SURF features, respectively, when a reduced image dataset was analyzed. In recent years, various machine learning algorithms like ANN, k-Nearest Neighbor, SVM, Decision Tree and Random Forest have been employed to classify multiclass human skin disease, and ANN has shown the best performance among the chosen algorithms [28]. Several approaches reported in literature are based on the data uncertainty for computer-aided diagnosis systems based on General Type-2 Fuzzy Logic [29,30,31].

Two deep learning methods to perform skin lesion segmentation, feature extraction and classification were proposed [32]. They were correlated into two steps: in a first step, segmentation and a coarse classification were performed, and in the next step, a refinement of the coarse classification results was done by distance heat-map computation. In another paper, GLCM and color features of the lesion were combined and further used to train a Multilayer Feedforward Artificial Neural Network [33] for skin cancer detection. An accuracy of 93.7% for melanoma detection was reported for a total number of 206 images (119 melanoma and 87 non-melanoma type). In addition, computational tools to extract and learn high-level features automatically from raw images such as Convolutional Neural Networks (CNNs), fully convolutional residual network (FCRN), Google’s Inception v4 CNN, VGGNet Convolution Neural Network or deep residual networks (ResNets) have drawn researchers’ attention in recent years for melanoma detection and classification [34,35,36,37]. Kaymak et al. [36] used four FCNs, i.e., FCN-AlexNet, FCN-8s, FCN-16s and FCN-32s, in order to segment the skin lesions in images belonging to the ISIC 2017 dataset. They trained the proposed model with the ISIC 2017 dataset using 2000 RGB images having different resolutions. The performance accuracy ranged from 0.932 to 0.939 and the elapsed time was from 176 min (for FCN-AlexNet) to 508 min (for FCN-32s). Bakheet and Al-Hamadi [37] proposed a fully automated ANN for real-time melanoma detection using Gabor-based entropic features as highly discriminative descriptors for skin lesions. A Multilevel Neural Network (MNN) with an improved backpropagation algorithm provided an accuracy of 97.50%, sensitivity of 100% and specificity of 96.87% when 200 8-bit RGB dermoscopic images of melanocytic lesions from the PH2 database were analyzed. However, these deep learning tools do not provide information about how the meaningful features were selected and; also, they require large training sets.

## 3. Materials and Methods

Our study set is composed of 1095 dermoscopic and non-dermoscopic images for skin lesions (i.e., melanoma, dysplastic nevus and regular nevus) collected from four databases (denoted B1 to B4); their properties are specified in Table 1. The selection of these databases was driven according to the analysis performed by Pérez et al. [38]. They show that a high variability in skin lesion images exists, which underlays the intricacy of the skin cancer diagnosis problem when using these public databases. This is due to significant feature overlapping between the different lesion classes.

We devoted our study to these lesions because we intended to reflect the data diversity encountered in daily clinical practice and to overcome the difficulty in differentiating skin lesions prone to a high degree of misdiagnosis like dysplastic nevus.

In the proposed approach, the class imbalance does not influence the classification accuracy.

We used 765 images to train the model, 165 cases for design validation and 165 images to test and report results. In addition, for each image we selected a subset of four asymmetry features. Thus, the proposed ANN model is trained using 3060 input data (4 × 765 matrix), validated and tested with 600 input data each (4 × 150 matrix). The ANN model predicts three possible clinical classes for skin lesion: melanoma, dysplastic nevus and regular nevus.

**Figure 1:**
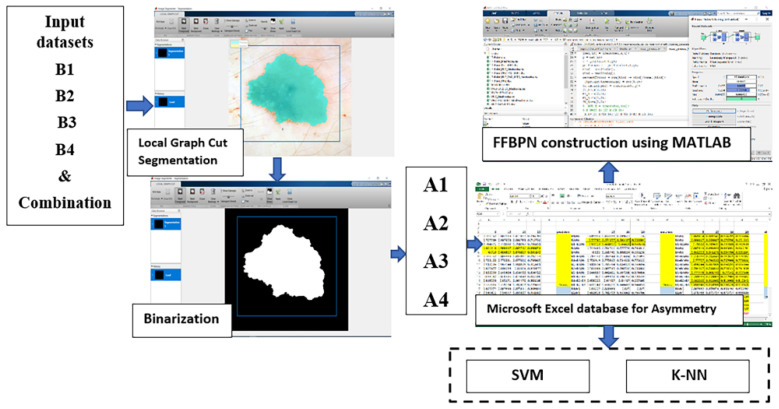
Overall proposed methodology for skin lesion recognition and classification.

The proposed process of skin lesion recognition and classification is presented in Figure 1.

### 3.1. Feedforward Back Propagation Network (FFBPN) Architecture

ANNs consist of parallel systems for information processing from nodes to nodes through variable weights. FFBPNs are a type of ANN that use a back propagation algorithm as a supervised learning method. The feedforward step forward propagates the external input information from the input nodes to the output. The backward phase computation updates the internal weights of the input data and calculates errors to produce an expected output data. The proposed model is used as a classification problem. Factors such as number of inputs, output units, size of the training dataset, complexity of the learning stage, activation and training functions impacts the optimum number of hidden neurons [43]. Usually, the over-training of an ANN increases with the number of hidden neurons and number of training runs.

The proposed models have the same activation functions, use the same training algorithm with the same default training parameters but have variable inputs and outputs. The network is trained with Levenberg-Marquardt Backpropagation (LMBP) algorithm, which minimizes functions that are sums of squares of some nonlinear functions, as it is the most over-training resilient. The mean square errors (MSE) of training, validating and testing are used to assess the network’s architectures. It is helpful to prevent the algorithm from frequently getting stuck in a local minimum of the error function.

The classification is done using four asymmetry features as inputs to ANNs. We face a multiclass classification problem with three classes as follows: (100) for nevus, (10) for melanoma and (1) for dysplastic nevus.

Optimizing the proposed ANNs means having the most suitable architecture for multiclass medical diagnosis decision making. We investigate architectures with 8, 12, 16 and 20 hidden neurons in the hidden layer, for each asymmetry feature. Through this approach we search for a net that better generalizes than memorizes patterns in the data. 

### 3.2. Data Collection

Based on the opinion of experts [44], the asymmetrical shapes displayed by skin lesions were chosen as an intuitive indication of its malignant potential. Thus, four different ways to acquire data about this parameter were proposed. First, the images were segmented using the Local Graph Cut in the Image Segmenter tool of MATLAB. Then, they were transformed into binary images (Figure 1).

Asymmetry Index *A*1—the axes of the ellipse circumscribing the object are determined by finding the center of mass of the image and then by rotating the major and minor axes until they become parallel to the Cartesian axes. The object is then folded along the main axes, and the two parts, when folded, lead to maximum overlap, computed with the XOR logic function:

(1) $$A1=\frac{\Delta A^{minor}}{AL}+\frac{\Delta A^{major}}{AL}$$

where *AL* denotes the area of the lesion and $\Delta A^{minor}$ and $\Delta A^{major}$ are the non-overlapping parts’ area obtained with the XOR function.

Asymmetry Index *A*2—another form for the asymmetry feature can be derived from the above data, as follows:

(2) $$A2=\frac{\min\left(\Delta A^{minor},\;\;\Delta A^{major}\right)}{AL}$$

Asymmetry Index *A*3—another approach that highlights the quadrants of an object that are not similar is so-called quadrant asymmetry. The proposed version is adapted from [45,46]. The object is divided into four quadrants and the centroids and center of masses of object and each quadrant are established. The asymmetry feature for the quadrant *i* is computed as

(3) $${ƛ}_{i}=\left(\frac{AL}{\Delta A_{i}}\right)\left(\frac{{\mathrm{dD}}_{i}}{{\mathrm{mM}}_{i}}\right),\;i=\bar{1,4}$$

where *AL* is the area of the lesion and $\Delta A_{i}$ is the area of the lesion in a particular quadrant *i*. ${\mathrm{dD}}_{i}$ denotes the distances from the centroids of the lesion within quadrant *i* to the centroid of the whole lesion and ${\mathrm{mM}}_{i}$ are the distances from the center of mass within the quadrant *i* to the center of mass of the whole lesion. An averaging operation over the four-quadrant asymmetry features $\;{ƛ}_{i}$ obtains the quadrant asymmetry  A3.

Asymmetry Index *A*4—the number of an object’s foreground pixels are projected over the Ox and Oy axes [47], and two histograms are generated. Horizontal *H* (*i*) and vertical *V* (*j*) histograms of the pixels’ projections are built by considering the number of ‘1′ pixels from each bin in both *x* and *y* directions.

(4) $$H\left(i\right)=\sum_{j=0}^{m-1}P\left[i,j\right],\;0<i<N,\mathrm{and}\;V\left(j\right)=\sum_{i=0}^{n-1}P\left[i,j\right],\;0<j<M,$$

where *H*(*i*) and *V*(*j*) store the foreground pixels in each row and column, respectively. P [*i*, *j*] is the pixel value at (*i*, *j*) and *N* is the width and *M* is the height of the binary image. Both histograms are compared using the correlation coefficient. This coefficient is the asymmetry from histogram projections feature.

(5) $$A4=\frac{\sum_{i=1}^{N}\left(H\left(i\right)-H^{\prime }\right)\left(V\left(i\right)-V^{\prime }\right)}{\sqrt{\sum_{i=1}^{N}{\left(H\left(i\right)-H^{\prime }\right)}^{2}}\sqrt{\sum_{j=1}^{M}{\left(V\left(i\right)-V^{\prime }\right)}^{2}}}$$

where *H*′ and *V*′ are the mean pixel values in the image. The more asymmetric the lesion shape, the lower the correlation value.

### 3.3. FFBPN Model Development

The proposed model is developed by using MATLAB 2018a (The MathWorks, Natick, MA, USA). The training function “trainlm” is performed using the Levenberg-Marquardt backpropagation to compute the Jacobian matrix of the performance function, respecting the weight and bias variables.

The overall data contains four parameters as system inputs and three output variables. The system inputs/control variables are the asymmetry indexes *A*1, *A*2, *A*3 and *A*4. The output variables are (100) or nevus, (10) for melanoma and (1) for dysplastic nevus.

The model training starts with a random initial population. After the training stage is completed, 15% of the data are used for validation and 15% of the data for testing.

**Figure 2:**
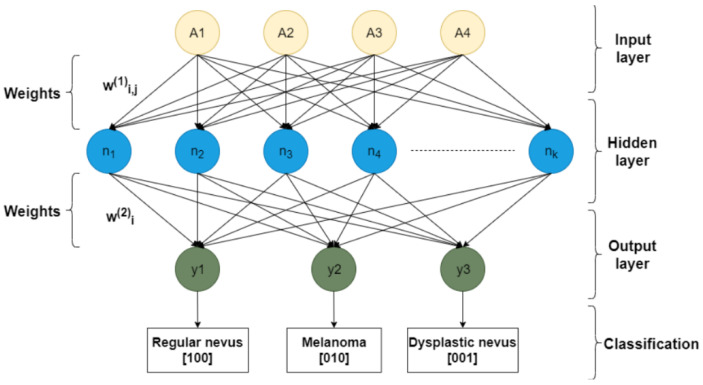
The FFBPN architecture (k = 8, 12, 16, 20 neurons). $w_{i,j}^{\left(1\right)}$ denotes the weight that connects the output of the *j*-th neuron of the input layer to the input of the *i*-th neuron of the hidden layer, and $w_{i}^{\left(2\right)}$ is the weight that connects the *i*-th hidden neuron to the output layer neuron.

The proposed neuronal network architecture is presented in Figure 2.

**Figure 3:**
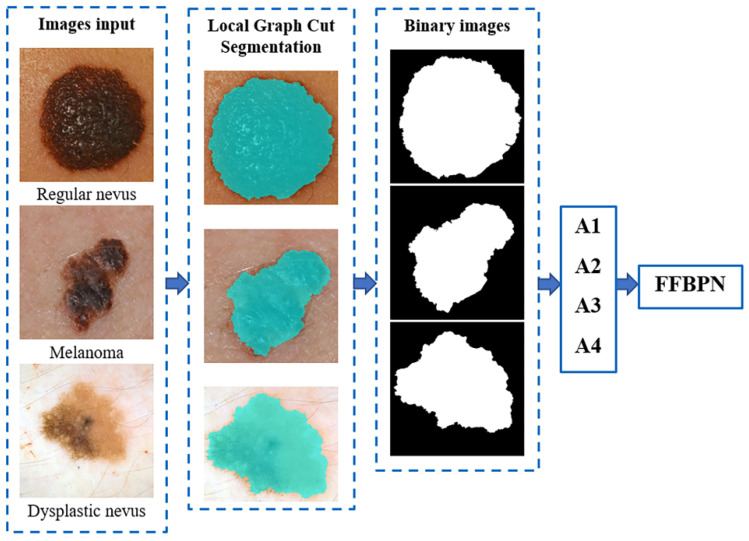
Skin lesion segmentation, the binary image detected, and the lesion asymmetry measures derived.

The skin lesion segmentation method is presented in Figure 3. The Image Segmenter app in Matlab was employed for interactive segmentation using the Graph Cut algorithm. Graph Cut segments objects within the boundaries of the ROI called scribbles, for a correct identification of the foreground and background. This method is an improvement of the methods proposed in our earlier work [46]. After segmenting the skin lesion, we compute the asymmetry, which is the A feature of the ABCDE rule, using the methods presented in the Section 3.2.

One of the research questions here is how many neurons will be in the hidden layer? The number of hidden neurons must be carefully considered as it has a huge impact on the final output. Shortcomings like overfitting or underfitting are closely related to the number of hidden neurons and lead to distortion of the predicted results. In addition, an inadequate number of hidden neurons will increase the training computation time and can affect the training stage. The second research question is related to the input data selection: what are the representative asymmetry features that allow the ANN to learn and classify lesions properly?

As there is no well-established theory to find out how many hidden neurons are needed for an accurate prediction or which are the meaningful features to win end-user trust, there are various rules of thumb to make this prediction, based on the number of input and output nodes, input samples and number layers. Ke and Liu [48] proposed the following formula to determine the number of hidden neurons: $N_{h}=\left(N_{in}+\sqrt{N_{p}}\right)/L$, where $N_{in}$ and $N_{p}$ denote the number of input nodes and input samples, respectively, and *L* is the number of hidden layers. In the above-mentioned study, $N_{in}=4$, $N_{p}$ varies from 170 to 765, *L* = 1 and the resultant number of hidden nodes $N_{h}$ spans from 14 to 31. In our research, we investigate the number of hidden nodes varying from 8 to 20, with a step of 4, and we used the general rule that the performance of the proposed architectures is assessed by using the area under the ROC curve (*AUC*) along with performance metrics such as sensitivity (important when identifying the total positives), accuracy (proportion correctly classified), precision (positive predictive value) and Dice coefficient (penalizes for false positives) [49].

$$\begin{array}{c} Sensitivity=\frac{TP}{TP+FN},\\ Accuracy=\frac{TP+TN}{TP+FP+FN+TN}\\ Precision=\frac{TP}{TP+FP}\\ DICE=\frac{2TP}{2TP+FP+FN}\\ AUC=1-\frac{1}{2}\left(\frac{FP}{FP+TN}+\frac{FN}{FN+TP}\right) \end{array}$$

where these metrics are extracted from the confusion matrix and are the true positives (*TP*), the false positives (*FP*), the true negatives (*TN*) and the false negatives (*FN*). In addition, the prediction performance of the proposed models is assessed based on minimum MSE on the test group. The learning rate was set to 0.01, and the number of iterations to 1000.

## 4. Results and Discussion

The asymmetry features are fed as input to the neural network. In order to find out the optimal model, sixty FFBPN architectures were tested for different input datasets, provided by four datasets and all their possible combinations and for two output dimensions (two-class and three-class decision problems). The size of the input dataset varies as the asymmetry data have different patterns provided by different benchmark datasets. There are two output dimensions, as the B1 and B2 datasets have three classes, while B3 and B4 datasets have only two classes. The number of neurons in the hidden layers was changed from 8 to 12, then to 16, and finally to 20. The ANN performance is considered for the following scenario: 70% of the data is training data, 15% is used for validation and 15% for verification.

To avoid the ANN memorizing the answer rather than generalizing the data patterns, each analyzed net was trained and tested using various input dataset sizes and data patterns (asymmetry feature $A_{i},\;i=\bar{1,\;4}$, provided by benchmark datasets $B_{j},\;j=\bar{1,\;4}$ and all their possible combinations). All possible combinations are used so that the number of input data is progressively increased as the number of benchmarking datasets providing the input feature increases. After the runs were completed, the performance of each feature was presented in terms of standard metrics: sensitivity, accuracy, precision, *AUC* and *Dice* coefficient metrics. *AUC* combines the sensitivity and specificity of the classifier.

Unlike most works, which have focused on the exploitation of the deep learning in melanoma detection mainly through the understanding of the importance of the training step in the global net’s management, such as the feature extraction and/or a classification (i.e., melanoma vs. benign) task, we use a FFBPN to investigate the redundancy of the shape asymmetry, as an efficient feature that can be exploited for the differentiation between benign and malign skin lesions during the classification process. To accomplish this task, we train the net on the four extracted asymmetry features based on skin lesion segmentation results.

**Figure 4:**
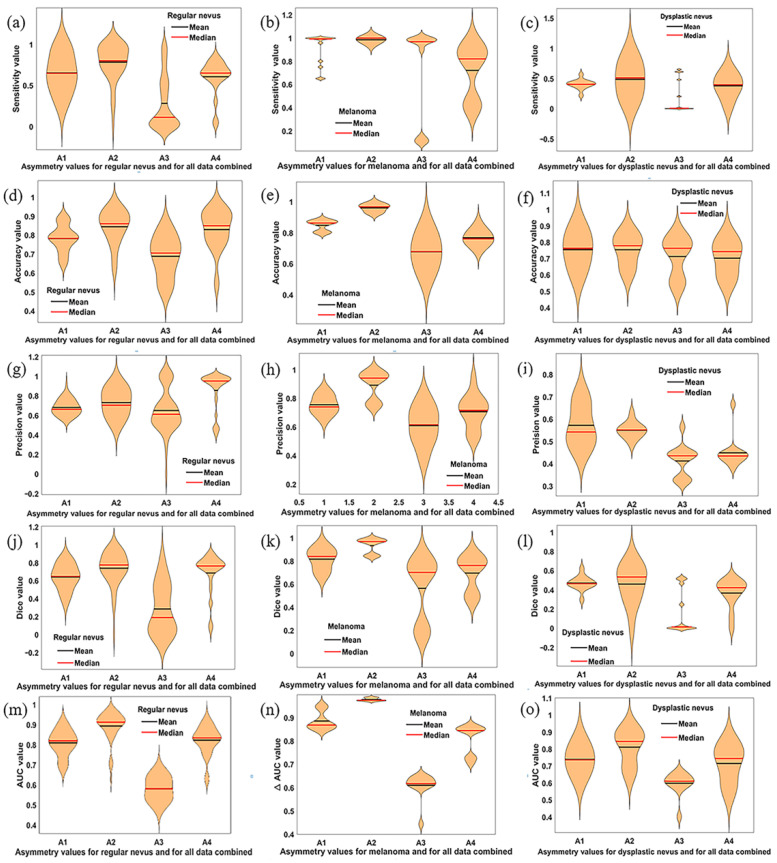
Violin plots showing the statistical distribution of sensitivity, accuracy, precision, *Dice* coefficient and *AUC* metric values on test set and for four asymmetry features (*A*1, *A*2, *A*3 and *A*4). Data provided for all ANN models and from all datasets were combined. The left column displays data for regular nevus. The middle column presents data for melanoma. The right column shows results for dysplastic nevus. The first row (**a**–**c**) contains the sensitivity values, the second row (**d**–**f**) is for accuracy, 3th row (**g**–**i**) displays the precision, 4th row (**j**–**l**) is for *Dice* and 5th row (**m**–**o**) is for *AUC*, respectively. Mean and median values for each approach are displayed. Significant differences detecting asymmetry comparisons are shown.

Figure 4 shows the violin plots for all metrics under consideration and for all experimental conditions.

In Figure 4, we demonstrate that all the performance metrices for *A*2, with their corresponding mean and median values, reach the best results. Among these results, data for the melanoma show an excellent classification performance. The *A*1 and *A*4 features indicate a good classification performance but lower than *A*2. One can observe in this figure that the *A*3 asymmetry feature led to the poorest performance results for all analyzed classes. The overall performance measures for each asymmetry feature were calculated and are indicated in Table 2.

The FFBPN model correctly classified 308 out of 319 melanomas in the test set, yielding an overall accuracy rate of 96.7% for the *A*2 asymmetry feature. In addition, the sensitivity or true positive rate, which determines the fraction of melanomas correctly identified, is one (100%) for *A*2. Moreover, the net provides for the same *A*2 feature a *Dice* coefficient of 0.969 and an *AUC* of 0.975 for melanoma classification. For dysplastic nevus, the results of sensitivity, *Dice* coefficient and *AUC* are much better for *A*2 than the other three asymmetry features. Similarly, the sensitivity, accuracy, *Dice* coefficient and *AUC* for regular nevi indicate a good performance for *A*2. The least sensitive and performant asymmetry parameter is *A*3 or so-called quadrant asymmetry.

**Figure 5:**
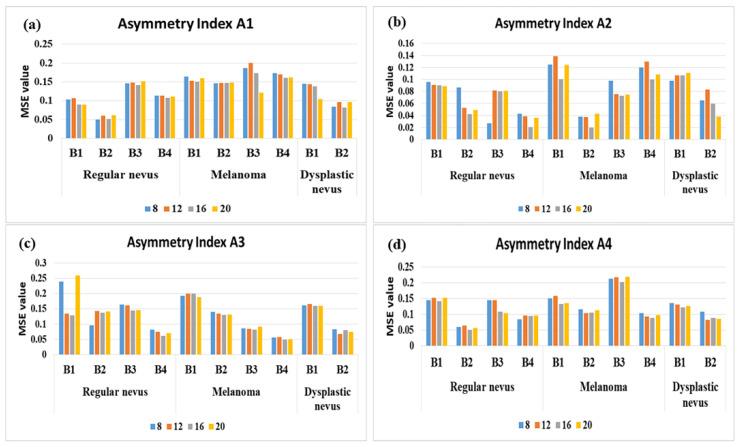
Performance (MSE for test group) for the proposed architectures for the optimal selection of number of hidden neurons. (**a**) Asymmetry Index *A*1; (**b**) Asymmetry Index *A*2; (**c**) Asymmetry Index *A*3; and (**d**) Asymmetry Index *A*4. (B1) denotes 7-Point database; (B2) is PH2; (B3) is MED-NODE and (B4) is PAD-UFES-20.

As the performance of the selected model is strongly influenced by the number of hidden nodes, we operated the model for a number of hidden nodes varying from 8 to 20, with a step of 4. To find an optimal model, we started with a high-capacity model (8 nodes), and then we adjusted the model for improvement in the validation metrics. Figure 5 displays the ANN architectures that highly conform to the experimental data for the asymmetry feature selection and according to the hidden neurons number criterium. As expected, the performance of the classification is driven by the number of hidden nodes being different from one asymmetry feature to other. Overall, we found out that net with 16 hidden neurons stabilizes the error and minimizes the overfitting. In other words, it optimizes the learning capacity and performs well both on the training and testing data.

The best FFBPN testing result found is an architecture with 16 hidden neurons, which provided an average accuracy of 96.7% and MSE of 0.0203 for *A*2/melanoma/B2. It is noteworthy that *A*1, *A*3 and *A*4 asymmetry features have a relatively equal overall performance, with MSE (*A*1) = 0.127, MSE (*A*3) = 0.125 and MSE (*A*4) = 0.120, while MSE (*A*2) = 0.077. The correlation between the ANN performance and number of hidden neurons indicates the following MSE values: for 8 neurons in the hidden layer, MSE = 0.116; for 12 neurons, MSE = 0.115; for 16 neurons, MSE = 0.106; for 20 neurons, MSE = 0.111.

On average, in term of asymmetry feature relevance, PH2 outperforms the other datasets in terms of MSE for *A*1 and *A*4, for regular nevus class. PH2 and PAD-UFES-20 outperform the rest of the datasets for *A*2 for regular nevus and melanoma classes. PAD-UFES-20 is relevant for *A*3 and melanoma class. The 7-Point and MED-NODE databases have a modest performance in terms of asymmetry feature relevance. The general assessed performance of the FFBPNs was higher on dermoscopic images (B1, B2 and B4 datasets), while the MED-NODE dataset (B3 dataset) contains low-resolution non-dermoscopic images shot with common digital cameras. However, all four datasets perform better for the number of hidden neurons equal to 16.

The skin lesion classification was also performed using probabilistic support vector machine (SVM) and k-nearest neighbor (k-nn) classifiers. A comparative analysis of the proposed lesion classification method (FFBPN with 16 hidden neurons), with the performance results of the two different methods, SVM and k-nn, for *A*2 is given in Table 3.

The proposed ANN architecture achieves the best accuracy as compared to other best classification methods. The neural network (FFBPN) was selected based on the best accuracy performance (0.860; 0.967; 0.778). The achieved recognition accuracy of SVM and k-nn is in the range (0.776; 0.881; 0.808) and (0.705; 0.764; 0.786), respectively. As overall performance of this multiclassification task, FFBNP achieved the higher classification performance for melanoma and regular nevi, while the performance of this classifier was slightly decreased for dysplastic nevi.

To completely assess the proposed approach, some reported data on the classification performance previously published on this topic are discussed. Ashfaq et al. [33] associated the ABCD features with GLCM parameters and trained a Multilayer Feedforward Artificial Neural Network. However, in the reported results, there was no noticeable enhancement as compared to using ABCDE features alone. The asymmetry in this work is the same with *A*1 in our work. For a net architecture using the ABCDE features as input, with one hidden layer and 14 hidden neurons, they reported an accuracy of 93.7% and a sensitivity of 95.8%. In addition, they used 206 images from DermIS and DermQuest datasets. In our paper, the performance is better in terms of sensitivity (100%) and accuracy (96.7%) for *A*2 asymmetry. However, in our work, for *A*1 asymmetry a sensitivity of 99.2%, and an accuracy of 86.2% are reported. We used 1095 dermoscopic and non-dermoscopic images collected from four databases (7-Point database; PH2; MED-NODE and PAD-UFES-20). Moreover, in the referenced work, the accuracy did not improve when the number of hidden layers and neurons increased.

A two-class decision problem that embeds the logistic regression and a filtering mechanism for attribute relevance, in order to initiate the hidden nodes of a single-hidden layer feedforward neural network (SLFN), which is meant to prevent the overfitting phenomenon, is discussed in [50,51]. Both proposed models were checked using publicly available cancer datasets, with the declared goal of benchmarking the classification performance, reducing the number of features, increasing the computational speed and decreasing the computational cost. Belciug [51] reported that the filtered logistic SLFN (fLogSLFN) achieves accuracies between 64.70% and 98.66%, depending on the used dataset, and concluded that the proposed model performs just as well as other state-of-the-art models. The number of hidden neurons varied from 300 (for breast cancer, Duke, and lung cancer, Michigan, databases) to 500 (for breast cancer, Kent Ridge database). Belciug and Gorunescu [50] compared their adaptive single-hidden layer feedforward neural network (aSLFN) with a single-hidden-layer feedforward neural network trained by a backpropagation algorithm. The best accuracy (95.69%) was obtained with this setup. However, the proposed method functions only for two-class decision problems. The benchmarking analysis of our results proved that our algorithm has the same efficacy as other classifiers, which, for example, use only a single-hidden-layer feedforward neural network. However, unlike many existing models, which are binary classifiers, our model is a multiple decision classes classifier.

Popular classifiers, such as CNNs used to classify skin lesions into benign or malignant lesions based on various approaches, were presented in [52,53]. The parameter B in the ABCDE rule, i.e., the skin lesion border detection and border irregularity estimation, was used for the input features for a CNN, which uses binary classification (i.e., melanoma vs. non-melanoma) [52]. They reported that all the regular borders were identified correctly, and only three irregular borders were classified as regular; thus, an overall accuracy of 93.6% was obtained. In addition, sensitivity and specificity of 100% and 92.5%, respectively, were reported. This finding is very consistent with our results for the asymmetry *A*2, even if they focused on the border irregularity and our study is devoted to the non-overlapping parts’ area between the surface of the ellipse delineating the object and the area of the lesion. CNNs are used as binary classifiers that discriminate between benign or malignant lesions based on a novel regularizer technique proposed by Albahar [23]. The author reported an excellent average accuracy of 97.49%. In addition, the performance in terms of *AUC*-ROC for the model was 98.3% during the validation step. These findings indicate that the model effectively discriminated between malignant and benign lesions. However, CNNs are so-called “black-box models”, and there is no information on how these models provided the final predictions. This makes the identification of the weaknesses and strengths of different net architectures very difficult. Moreover, the performance of CNNs is slightly lower than the feed-forward network. This finding is consistent with [54].

The above results showed that a 16 hidden neurons FFBPN model is suitable for melanoma diagnosis when the asymmetry feature *A*2 is used as input data. There are other analyzed models with a similar prediction accuracy, so a further task would be a sound investigation of the performance of the FFBNP models, as the skin lesion structures encountered in analyzed images are complex in shape, have ambiguous boundaries and vary in size. Bano et al. [55] analyzed the performance of both backpropagation neural network (BNN) and an auto-associative neural network (AANN) in terms of number of neurons in the hidden layers. They reported an overall accuracy of 90.2% for BNN and 81.2% for AANN, which increased with the number of hidden neurons; they concluded that the number of hidden layers is an adjustment factor against over-fitting, but it cannot improve the result.

## 5. Conclusions

In this work, a sound investigation towards skin lesion recognition throughout the different datasets was carried out, with the declared goal of establishing the weaknesses and advantages of different FFBPN architectures in melanoma, dysplastic nevus and regular nevus classification. The experimental analysis assessed 60 FFBPNs on four public image datasets for four asymmetry features. The image database contained a sufficiently large number of images. This includes both dermoscopic and non-dermoscopic images. In the proposed approach, the class imbalance does not influence the classification accuracy, as it was demonstrated using different input datasets provided by four datasets and all their possible combinations. We found that there was a large variation in terms of performance measures plus overall performance of the network, which was largely between the dermoscopic and digital image. In addition, we used the FFBPN architectures to investigate the redundancy of the shape asymmetry as an efficient feature in differentiation benign and malign skin lesions during the classification process.

Results show a high variability in skin lesion images and underlaid the complexity of the melanoma diagnosis problem when using these publicly available databases due to the feature overlapping between the investigated lesion classes. Moreover, the results clearly indicate that the best classification is obtained when the selection of most discriminant features is properly performed. Our experimental results on multiple benchmark datasets show that the asymmetry *A*2 is a meaningful feature for skin lesion classification, and FFBPN, with 16 neurons in the hidden layer model, achieved better classification with the three classes task. This FFBPN architecture is validated with an MSE of 0.106. In addition, it was observed that the FFBPN is a better predictor than SVM and k-nn classifiers, and it can significantly influence the predictive performance.

This assistive tool belongs to the Internet of Medical Things (IoMT) and is used to aid dermatologists during the decision-making process.

For our future research, we plan to develop an end-to-end mobile application system, able to inspect a lesion in real time using a mobile phone camera. Furthermore, future works may also involve the investigation of the applicability of the proposed method to recognize other pathologies of the skin or to investigate medical images like computed tomography, magnetic resonance imaging, etc.

## Figures and Tables

**Table 1:** Class distribution statistics of benchmark datasets.

Datasets	No. of Images	No. of Classes	No. of Attributes	No. of Nevus/ Melanoma/ Atypical Nevus	Image Type
7-Point (B1)	439	3	4	68/297/74	Dermoscopic [39]
PH2 (B2)	197	3	4	80/40/77	Dermoscopic [40]
MED-NODE (B3)	170	2	4	100/70/0	Non-dermoscopic [41]
PAD-UFES-20 (B4)	289	2	4	241/48/0	Dermoscopic [42]

**Table 2:** Performance measures for each asymmetry feature and for all models (the bold values indicate the highest performance).

Sensitivity				
	*A*1	*A*2	*A*3	*A*4
Regular nevus	0.648	0.801	0.111	0.651
Melanoma	0.992	**1**	0.968	0.820
Dysplastic nevus	0.404	0.511	0.006	0.397
**Accuracy**				
	*A*1	*A*2	*A*3	*A*4
Regular nevus	0.783	0.860	0.705	0.849
Melanoma	0.862	**0.967**	0.678	0.760
Dysplastic nevus	0.762	0.778	0.762	0.742
**Precision**				
	*A*1	*A*2	*A*3	*A*4
Regular nevus	0.661	0.702	0.610	**0.950**
Melanoma	0.739	0.941	0.615	0.715
Dysplastic nevus	0.542	0.551	0.435	0.434
**Dice coefficient**				
	*A*1	*A*2	*A*3	*A*4
Regular nevus	0.648	0.775	0.190	0.764
Melanoma	0.841	**0.969**	0.702	0.762
Dysplastic nevus	0.464	0.532	0.012	0.422
**AUC**				
	*A*1	*A*2	*A*3	*A*4
Regular nevus	0.819	0.913	0.581	0.834
Melanoma	0.868	**0.975**	0.617	0.845
Dysplastic nevus	0.736	0.845	0.610	0.744

**Table 3:** Comparative analysis between the performance results of the skin lesion classification for *A*2 asymmetry feature on all used datasets (the bold values indicate the highest performance).

Classifier	*Accuracy*	*Sensitivity*	*Precision*
SVM			
Regular nevus	0.776	0.711	0.712
Melanoma	0.881	0.898	0.765
Dysplastic nevus	0.808	0.662	0.612
k-nn			
Regular nevus	0.705	0.693	0.726
Melanoma	0.764	0.893	0.812
Dysplastic nevus	0.786	0.692	0.616
FFBPN (16 hidden neurons)			
Regular nevus	0.860	0.801	0.702
Melanoma	**0.967**	**1**	**0.941**
Dysplastic nevus	0.778	0.511	0.551

## Data Availability

Not applicable.
